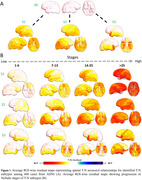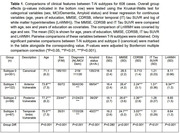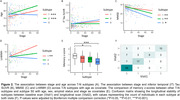# Event‐based modeling of T‐N mismatch reveals distinct spatio‐temporal trajectories of subtypes with likely co‐pathology in Alzheimer’s disease

**DOI:** 10.1002/alz.094054

**Published:** 2025-01-09

**Authors:** Xueying Lyu, Paul A. Yushkevich, Stanislau Hrybouski, Yue Li, Michael Tran Duong, Long Xie, Laura E.M. Wisse, Robin de Flores, David J Irwin, Bradford C. Dickerson, Ilya M. Nasrallah, Sandhitsu R. Das, David A Wolk

**Affiliations:** ^1^ University of Pennsylvania, Philadelphia, PA USA; ^2^ Perelman School of Medicine, University of Pennsylvania, Philadelphia, PA USA; ^3^ Department of Clinical Sciences Lund, Lund University, Lund, Lund Sweden; ^4^ Normandie Univ, UNICAEN, INSERM, U1237, PhIND Physiopathology and Imaging of Neurological Disorders, NeuroPresage Team, GIP Cyceron, Caen France; ^5^ Massachusetts General Hospital, Charlestown, MA USA; ^6^ Department of Radiology, University of Pennsylvania, Philadelphia, PA USA; ^7^ Penn Image Computing and Science Laboratory (PICSL), University of Pennsylvania, Philadelphia, PA USA; ^8^ Center for Neurodegenerative Disease Research, Perelman School of Medicine, University of Pennsylvania, Philadelphia, PA USA

## Abstract

**Background:**

The heterogeneity of Alzheimer’s disease (AD) and lack of well‐validated markers of co‐pathologies present a substantial challenge for therapeutics. We previously found phenotypes defined by Tau (T) ‐ Neurodegeneration (N) discordance linked to non‐Alzheimer’s pathologies (e.g. TDP‐43, vascular disease). In this work, we aim to leverage T‐N mismatch for identifying distinct spatial‐temporal progression patterns of non‐AD pathologies.

**Method:**

We performed T‐N regression on 1040 scan pairs (n = 722 individuals) from ADNI, using cortical thickness (N) and 18F‐Flortaucipir uptake (T) in 20 bilateral cortical regions of interest. As in previous work, residuals were identified as canonical (T∼N), vulnerable (N>T) and resilient (N<T). Here, we apply SuStaIn, a phenotype discovery and stage inference algorithm, to standardized T‐N residuals in canonical and vulnerable cases (n=608), expecting the latter would reflect co‐pathologies.

**Result:**

Besides the "canonical" subtype (S0), SuStaIn identified three subtypes with distinct T‐N mismatch (N>T) progression profiles (Figure 1). Two exhibited different but progressively diffuse spatial patterns of T‐N mismatch — the anterior subtype (S1) starting from frontal, and the posterior subtype (S2) initiating from occipital/temporal regions. The third subtype (S3) exhibited temporal‐limbic mismatch patterns, with spreading to anterior limbic regions. The three mismatch subtypes had worse cognition, greater age and larger rate of amyloid positivity than canonical subtype (Table 1). SuStaIn‐identified stage was associated with age and worse cognition but not tau severity (Figure 2A‐C), indicating the stages do not represent AD progression. The anterior subtype had the largest white matter hyperintensity (WMH) volume, increasing with higher stages (Figure 2D), suggesting an association with vascular disease. The temporal‐limbic subtype demonstrated poorest memory performance (Figure 2E) and may be associated with TDP‐43 pathology, as suggested in our prior work. 90.1% with longitudinal scans did not change subtype or transitioned from canonical to mismatch subtypes (Figure 2F), supporting the stability of classifications.

**Conclusion:**

We identified distinct T‐N mismatch progression trajectories in AD, potentially reflecting progression of co‐pathologies. It is important to better define these groups in the context of anti‐amyloid therapies to better understand their effectiveness in populations with likely co‐pathology and whether these mismatch trajectories driven by non‐AD factors would continue independent of treatment.